# Supplementary material for: Dynamic interactions between epithelial skin cells and a sensory cavity sculpt the growing olfactory orifice
Source: Nat Commun. 2026 Jul 8;17:8452. doi: 10.1038/s41467-026-75307-y (PMC13478634; doi:10.1038/s41467-026-75307-y)
Supplement: Supplementary file 2 [file 41467_2026_75307_MOESM12_ESM.pdf]

nature portfolio

Peer Review File

Dynamic interactions between epithelial skin cells and a sensory cavity sculpt the growing olfactory orifice

Corresponding Author: Dr Marion Baraban

This file contains all reviewer reports in order by version, followed by all author rebuttals in order by version.

Version 0:

Reviewer comments:

Reviewer #1

(Remarks to the Author)
In this manuscript, the authors investigate mechanisms that control the development of the olfactory orifice in zebrafish. This work follows previous work by the group that indicates neurons in the olfactory placode use actomyosin contraction to pull on the overlying skin to open the olfactory orifice (Dev Cell. 2023; DOI: 10.1016/j.devcel.2023.02.001). Here, the focus is on understanding how growth and shape of the developing olfactory orifice (epithelial hole) is regulated. Using cutting-edge techniques in zebrafish, including tissue-specific perturbations, the authors find that orifice expansion is driven by growth of the underlying olfactory placode cavity and that orifice shape is generated via tight regulation of actomyosin contractility. Overall, the quality of the data presented is high and conclusions are supported by the results. The use of multiple experimental perturbations—genetic, pharmacological, and physical—is a strength of the work. The figures and movies are well organized and clear, and the schematics are very effective. Significant impacts of this work include the identification of tissue-tissue interactions and mechanical forces that govern the formation of the olfactory orifice; mechanisms that that may broadly apply to the regulation of gaps/holes in epithelial barriers. I have a few comments for the authors to consider:

Major points:
1) The quantification of phenotypes (e.g. cavity area, orifice area, etc.) is fairly consistent throughout the manuscript. However, in Fig. 4 the authors use orifice solidity to presumably describe orifice shape after observing “a wiggly orifice edge” in embryos with reduced myosin activity (Fig. 4e). This measurement should be defined/clarified in the text.

2) Two mutant lines (dnaaf1 and elipsa) are used to test whether cilia are involved in orifice formation. While loss of cilia function does not change orifice size, it appears that orifice shape is affected in both mutants (Fig. S2) suggesting a cilia-dependent mechanism regulating the orifice edge. Measuring orifice solidity in existing images would address this.

3) For reproducibility, it will be useful to indicate approximately how big of an area (or how many cells) of the placode (Fig. 3D) was ablated at 32 hpf to reduce placode size by ~25% by 48 hpf.

4) It should be clarified whether the Tg(uas:DNmyosin-Scarlet) line is the same as the Tg(UAS:mScarlet-DN-MYL9) line described in previous work (Dev Cell. 2023; DOI: 10.1016/j.devcel.2023.02.001). If so, it appears that expressing DN MyosinII in the skin (krt5 driver) is not having the same effect as previously described: “larger orifices and rosette cavities.” This should be addressed in the text.

Minor point:
1) I couldn’t find captions that describe what we are watching in the movies. As examples, it is not clear what the arrows point out in Movies 5 and 6.

Reviewer #2

(Remarks to the Author)
In this manuscript, Gordillo and colleagues characterize the mechanism promoting the expansion of the olfactory orifice in zebrafish. Epithelial opening and hole formation is a very poorly characterized morphogenetic process. The authors use here the olfactory orifice as a very potent system to study the mechanisms regulating the shape and size of the formation of

an orifice and how interaction between two tissue layers can help to shape it. Using a combination of quantitative live imaging, genetic and drug perturbations with laser ablation, the authors first show that orifice expansion mostly relies on skin cell elongation and shape changes, with a minor contribution of cell division. They then prove that cell radial intercalation and growth of the placode cells is an important driver of the expansion. Finally, by combining various perturbative experiments they show that while myosin activity is required in the placode for proper growth, it seems to have mostly a negative role in the skin. This is mostly suggested by the impact of ERK inhibition which reduces orifice size and promote 3D buckling of the placode, which can be rescued by reduction of tension in the skin.

Overall, I really enjoyed reading the article which is very well written, documented with very clean experiments with proper quantification, control and the combination of various perturbative approaches. As stated above, we know very little about morphogenetic processes driving orifice formation in vivo and the precise phenomenology provided in this manuscript is very valuable and should be of interest for a wide readership.

One main limitation at this stage is the lack of a clear working model (in the sense of “mechanical” model) outlining the main parameters regulating orifice size, and the relative contribution of tension in the skin and placode. Currently, the interpretation of the various results perturbing MyoII and ERK are difficult to reconcile in a simple morphogenetic model and I would encourage the authors to make more clear proposition to fit all the data together (I have some suggestions along this line, see below, though it could be that other working model fits better). Similarly, the morphological characterization of the orifice and cavity is limited by the characterization in 2D on max projections. While this is probably ok in the WT context where the shape is mostly planar, it may hide very relevant information in perturbed contexts where cavity might be more bended/curved. Providing systematic information on the 3D shape of the cavity/placode 3D surface (at least with lateral view, or even better, with a measure of the apical placode surface in 3D) would help to get an intuition of the mechanisms at play and disentangle issues related to placode growth versus placode planar expansion (which is probably the one affected by the skin mechanical state and the MyoII purse-string).

I will list my main suggestions below, which mostly require novel quantifications on existing data, and suggestions that would require 2 novel experiments (hopefully manageable with reasonable time investment).

Main suggestions :

1. As mentioned above, the manuscript at this stage does not provide a clear working model integrating the various results. This is particularly relevant when trying to integrate the various experiments perturbing MyoII in the skin or in the placode. Currently, the manuscript data suggest that :
i) Placode growth (through cell division and radial intercalation) is the main driver of orifice expansion
ii) Orifice expansion is slow down/balanced by the MyoII purse string at the skin interface
iii) Skin tension/MyoII mostly act as a limitant factor for placode planar expansion (but not necessarily its real surface, see my point on 3D measurement)
iv) MyoII depletion in the placode perturb its expansion.

In this framework, if the placode indeed push on the skin boundary and is constraiedn by the actomyosin pursestring, it should tend to be under pressure (which match the negative impact of laser ablation in the placode on final orifice size).

Could the authors use their ablation experiment in the placode (potentially ablating a large disc of cells) and check on short time scale whether this is associated with expansion or contraction of the empty area ? This would provides valuable information about the tensional state of the placode.

2. As mentioned above, the authors mostly use z projection to measure the cavity area. However this may poorly reflect the total number of cells and real surface of the cavity/placode if there is significant bending. Could the author provide some estimate of the real 3D surface of the cavity in all the contexts ? This will help to decouple the parameters impacting growth of the placode versus the context impacting its capacity to laterally expand (which probably relies also on the relative stifness/tension between skin and placode). If the 3D measurement is too tedious with the resolution they have, they should at least provide some lateral view and an estimation of the number of cells in the cavity.

3. Results in Figure 4d suggest that MyoII in the placode promotes orifice growth. Right now I see two ways to interpret this : either MyoII inhibition impact placode cell division and/or radial intercalation, or alternatively MyoII depletion affect the global stifness/bending modulus of the placode which cannot “push” anymore effectively on skin boundary (this is relevant only if the placode is indeed under pressure). Have the authors any way to estimate the number of placode cells in this context (inhibition of MyoII in the placode versus control) ? The fact that placode area remain the same (provided there is no change of bending) may suggest that division/intercalation are not significantly affected and would suggest that it is mostly the capacity to “push” that is limited.

4. The results on ERK are interesting but seems a bit preliminary to build a firm model. If the authors want to claim that ERK inhibits tension in the skin, they should then either compare the levels of actomyosin in DMSO and MEK inhibited skin and/or perform laser ablation in the skin to compare tension.

5. Similarly, do they have some estimation of the impact of ERK inhibition on placode growth/radial intercalation (as they did in the skin) ? Measuring real 3D surface of the placode would be quite essential here.

6. The authors do not really comment the phenotype of the orifice upon perturbation of MyoII in the skin (Figure 5d,d', comparing DMSO and DMSO krt5 DNMyoII). While their results on ERK and tissue bending suggest that skin tension can prevent the expansion of the orifice due to hypercontractility, it does not really fit with the absence of orifice area increase upon MyoII inhibition in the skin. Would the authors have some hypothesis to explain this ?

7. Along this line, intuitively skin tension may have opposing contribution to the expansion of the cavity, with bulk tension promoting expansion (provided that skin is indeed under tension) and the pursestring rather slowing down the expansion of the orifice. This may explain the surprisingly poor impact of MyoII inhibition in the skin on orifice area. Could the authors provide some speculation along this line ? (I suspect there is no way experimentally to specially block specifically MyoII in the purse string versus the bulk of the skin).

8. The authors characterize in figure 3 the expansion of the cavity through the number of small apical round area and the later inclusion of large cuboidal cells. However the surface in between the small apical area may also change and contribute to area expansion. Could the authors document also the evolution of spacing and/or density of the small apical area cells during development ? That would provide an exhaustive characterization of what contribute to placode growth. Moreover, could they provide some information on what stand in between those cells (other epithelial cells ? ECM ? mesenchymal cells ?). ?

Other minor points :

1. It is interesting to note that the recoil speed of the skin interface (Fig. 4b) does not seem to change with cavity area. It might be worth mentioning this point in the main text/discussion.

2. Could the authors provide measurement of the cavity perimeter and its smoothness in all the context perturbing MyoII (Figure 4) ? Right now, only the area is provided, but I suspect that perimeter is mostly unaffected (since number of bordering cells and border lengths are maintained). This would fit with a more irregular perimeter as well (hence increase perimeter other area ratio).

3. Regarding the contribution of cell division in the skin, while I do agree that the data of the authors suggest that cell division is not absolutely necessary for orifice growth, I note however that the initial cell geometry and apical area is very much different in the CDKN1B context. One cannot exclude that the mechanical state of the system from the start is quite different. The ideal experiment would be to block cell division temporally specifically at the time of orifice growth, but that is not possible with the skin specific genetic inhibition. I may suggest to mention this limitation somewhere the text.

Reviewer #3

(Remarks to the Author)
Clara et al. reported a study on the dynamic evolution of the olfactory orifice and the placodal cavity in zebrafish. They investigated how the size of these epithelial holes evolve over time and the regulating roles of cell contractility and ERK signaling. This research is in-depth, systematic and interesting, bridging a critical gap in existing literature of tissue hole dynamics. The manuscript is well written with clear logic and sufficient evidence. My concerns are provided below.

1. The placodal cavity, i.e., the apical side of placodal rosette cell, exhibited a dome-like shape in the three-dimensional space. However, the authors quantified only the growth dynamics of the projected area. In my opinion, it would be interesting to examine how the curvature of such a dome-like cavity evolves over time. Does it become more curved or flat as the cavity grows? In addition, it would also be interesting to quantify the shape of holes in the projected plane. I think these additional quantifications will strengthen this work.

2. The authors quantified the dynamic evolution of olfactory orifice and placodal cavity starting from 32 hpf with the orifice area ~100 um2 and cavity area ~ 200 um2, much larger than zero. Why there is no data before 32 hpf? (Small holes difficult to detect?) More discussion is required.

3. The clarity of the measurement method of the cavity area should be improved.

4. If the first or second neighbors are damaged, how will the tissue carry out self-rescue? What changes will occur in cell movement or morphogenesis?

5. Why the shape of the dorsal view is oval and has the characteristics of long axis and short axis? Is it related to the distribution of different types of cells? Or it is related to the distribution of actomyosin?

6. Just from the results of the cross-sectional diagrams (Fig. 1a), there are differences in the shapes of the cavity in the early (32-36 hpf) and late (48 hpf) stages. After interfering ERK pathway, the shape of the cavity is similar to that in the early stage (32-36 hpf). Does the effect of ERK pathway have a stage-specific functions? Does the ERK pathway have a minor effect in the initial expansion stage and a more significant effect in the later expansion stage?

Version 1:

Reviewer comments:

Reviewer #1

(Remarks to the Author)
The authors have address each of my comments in the revised manuscript. Clarifications and new experimental results have strengthened the paper, which will appeal to a broad audience.

Reviewer #2

(Remarks to the Author)
The authors have adressed all my concerns and significantly improved the manuscript, specially by clarifying the evolution of 3D shape/surface of the orifice/placode. The addition of the impact of skin MyoII inhibition on orifice growth is also compelling and streghten the conclusions. I am now fully supportive for pulbication of this important and rigorous study.

Reviewer #3

(Remarks to the Author)
I have no further comments and recommend its publication.

Open Access This Peer Review File is licensed under a Creative Commons Attribution 4.0 International License, which permits use, sharing, adaptation, distribution and reproduction in any medium or format, as long as you give appropriate credit to the original author(s) and the source, provide a link to the Creative Commons license, and indicate if changes were made.
In cases where reviewers are anonymous, credit should be given to 'Anonymous Referee' and the source.
The images or other third party material in this Peer Review File are included in the article’s Creative Commons license, unless indicated otherwise in a credit line to the material. If material is not included in the article’s Creative Commons license and your intended use is not permitted by statutory regulation or exceeds the permitted use, you will need to obtain permission directly from the copyright holder.
To view a copy of this license, visit https://creativecommons.org/licenses/by/4.0/

Response to reviewers

We would like to thank the reviewers for their thoughtful comments on the manuscript. As detailed below, we have addressed all of the points raised by the reviewers with additional data and modifications to the text and figures, which have substantially improved the manuscript and strengthened our initial conclusions. The changes made to the original manuscript appear in blue in the text of the article. We look forward to the reviewer's feedback on this revised manuscript.

Reviewer #1

In this manuscript, the authors investigate mechanisms that control the development of the olfactory orifice in zebrafish. This work follows previous work by the group that indicates neurons in the olfactory placode use actomyosin contraction to pull on the overlying skin to open the olfactory orifice (Dev Cell. 2023; DOI: 10.1016/j.devcel.2023.02.001). Here, the focus is on understanding how growth and shape of the developing olfactory orifice (epithelial hole) is regulated. Using cutting-edge techniques in zebrafish, including tissue-specific perturbations, the authors find that orifice expansion is driven by growth of the underlying olfactory placode cavity and that orifice shape is generated via tight regulation of actomyosin contractility. Overall, the quality of the data presented is high and conclusions are supported by the results. The use of multiple experimental perturbations—genetic, pharmacological, and physical—is a strength of the work. The figures and movies are well organized and clear, and the schematics are very effective. Significant impacts of this work include the identification of tissue-tissue interactions and mechanical forces that govern the formation of the olfactory orifice; mechanisms that that may broadly apply to the regulation of gaps/holes in epithelial barriers. I have a few comments for the authors to consider:

Major points:

1) The quantification of phenotypes (e.g. cavity area, orifice area, etc.) is fairly consistent throughout the manuscript. However, in Fig. 4 the authors use orifice solidity to presumably describe orifice shape after observing "a wiggly orifice edge" in embryos with reduced myosin activity (Fig. 4e). This measurement should be defined/clarified in the text.

The orifice solidity is the ratio between the orifice area and its convex hull area. A high solidity means that the edge of the structure has a smooth shape. This definition was already given in the "Materials and methods" section, and we now also added the definition in the main text (page 8), when we first mention solidity in the results.

2) Two mutant lines (dnaaf1 and elipsa) are used to test whether cilia are involved in orifice formation. While loss of cilia function does not change orifice size, it appears that orifice shape is affected in both mutants (Fig. S2) suggesting a cilia-dependent

mechanism regulating the orifice edge. Measuring orifice solidity in existing images would address this.

As suggested by the reviewer we have now included the analysis of orifice solidity in *dnaaf* and *elipsa* datasets (Supplementary Figure 3c'' and d''). We comment in the text about this new result page 8:
"We noticed a slight but significant reduction of orifice solidity in *elipsa* mutants compared to control siblings but no difference in *dnaaf* mutants (Supplementary Fig. 3c'', d''), suggesting that the presence of cilia, but not their beating, participates in the smoothening of the orifice edge."

We have also replaced the *dnaaf* $^{-/-}$ mutant image to better represent the new orifice solidity results (Supplementary Figure 3c).

3) For reproducibility, it will be useful to indicate approximately how big of an area (or how many cells) of the placode (Fig. 3D) was ablated at 32 hpf to reduce placode size by ~25% by 48 hpf.

As suggested by the reviewer, we now provide the average area of the ablated region in the "Materials and methods" section, page 18: "The mean ablated area was 191 μm^2 +/- 72 (quantified on 2 out of 3 experiments)."

4) It should be clarified whether the Tg(uas:DNmyosin-Scarlet) line is the same as the Tg(UAS:mScarlet-DN-MYL9) line described in previous work (Dev Cell. 2023; DOI: 10.1016/j.devcel.2023.02.001). If so, it appears that expressing DN MyosinII in the skin (krt5 driver) is not having the same effect as previously described: "larger orifices and rosette cavities." This should be addressed in the text.

We thank the reviewer for bringing up this important point, the line is the same indeed. We clarified that the two names refer to the same transgenic line, by adding the following precision in the "Materials and methods" (page 15): "*Tg(uas:DNmyosin-Scarlet)^{bps5}*, previously named *Tg(UAS:mScarlet-DN-MYL9)*[17], was used to inhibit myosin II activity."

As noticed by the reviewer, we previously observed an increased orifice area in *Tg(krt5:Gal4ERT; uas:DNmyosin-Scarlet)* embryos at an earlier stage, just after the orifice has opened (reference 17). Here, we did not detect such a phenotype at 48 hpf (Fig. 5d, d'). However, during the course of the revisions we performed additional experiments using the new *Tg(krt4:KalTA4)* driver made for this study, showing robust and strong skin expression. We observed an increased orifice area in *Tg(krt4:KalTA4; uas:DNmyosin-Scarlet)* embryos at 48 hpf, coupled to an increased cavity area, reinforcing the idea that actomyosin contractility in the skin counteracts the growth of the olfactory orifice and that this exerts a mechanical retroaction on the cavity. We think that these differences are due to differences in the levels of Gal4-mediated expression between the two driver lines at the stages analysed in this study. We present these new results in Supplementary Fig. 5d and in the text page 11, along with a note to compare with the results previously obtained with the *Tg(krt5:Gal4ERT)* line:

“Consistent with the idea that skin actomyosin contractility counteracts orifice expansion and that this exerts a mechanical retroaction on the cavity, skin-specific overexpression of dominant-negative Myosin II with the new *Tg(krt4:KalTA4)* driver made for this study, exhibiting strong and robust expression in the periderm, resulted in increased orifice and cavity areas at 48 hpf (Supplementary Fig. 5d). However this effect was not observed with the *Tg(krt5:Gal4-ERT)* driver at 48 hpf (Fig. 5d, d’) while it had been previously detected at an earlier stage (34 hpf), just after orifice opening[17]. This could be explained by differences in the levels of Gal4-mediated expression between the two driver lines during the initial expansion phase.”

Minor point:

1) I couldn’t find captions that describe what we are watching in the movies. As examples, it is not clear what the arrows point out in Movies 5 and 6.

We apologize for not having provided the movie captions in the initial version of the article. They can now be found in the section “Supplementary Movie legends”, page 33.

Reviewer #2

In this manuscript, Gordillo and colleagues characterize the mechanism promoting the expansion of the olfactory orifice in zebrafish. Epithelial opening and hole formation is a very poorly characterized morphogenetic process. The authors use here the olfactory orifice as a very potent system to study the mechanisms regulating the shape and size of the formation of an orifice and how interaction between two tissue layers can help to shape it. Using a combination of quantitative live imaging, genetic and drug perturbations with laser ablation, the authors first show that orifice expansion mostly relies on skin cell elongation and shape changes, with a minor contribution of cell division. They then prove that cell radial intercalation and growth of the placode cells is an important driver of the expansion. Finally, by combining various perturbative experiments they show that while myosin activity is required in the placode for proper growth, it seems to have mostly a negative role in the skin. This is mostly suggested by the impact of ERK inhibition which reduces orifice size and promote 3D buckling of the placode, which can be rescued by reduction of tension in the skin.

Overall, I really enjoyed reading the article which is very well written, documented with very clean experiments with proper quantification, control and the combination of various perturbative approaches. As stated above, we know very little about morphogenetic processes driving orifice formation in vivo and the precise phenomenology provided in this manuscript is very valuable and should be of interest for a wide readership.

One main limitation at this stage is the lack of a clear working model (in the sense of

“mechanical” model) outlining the main parameters regulating orifice size, and the relative contribution of tension in the skin and placode. Currently, the interpretation of the various results perturbing MyoII and ERK are difficult to reconcile in a simple morphogenetic model and I would encourage the authors to make more clear proposition to fit all the data together (I have some suggestions along this line, see below, though it could be that other working model fits better). Similarly, the morphological characterization of the orifice and cavity is limited by the characterization in 2D on max projections. While this is probably ok in the WT context where the shape is mostly planar, it may hide very relevant information in perturbed contexts where cavity might be more bended/curved. Providing systematic information on the 3D shape of the cavity/placode 3D surface (at least with lateral view, or even better, with a measure of the apical placode surface in 3D) would help to get an intuition of the mechanisms at play and disentangle issues related to placode growth versus placode planar expansion (which is probably the one affected by the skin mechanical state and the MyoII purse-string).

We thank the reviewer for these insightful comments. As detailed below, to address these points we (i) modified the text to clarify the mechanical model, (ii) performed additional experiments that complement and reinforce this model, and (iii) carried out new analyses to evaluate the surface and shape of the placodal cavity in 3D, which brought new information on the mechanisms driving the coordinated growth and morphogenesis of the cavity and orifice structures.

I will list my main suggestions below, which mostly require novel quantifications on existing data, and suggestions that would require 2 novel experiments (hopefully manageable with reasonable time investment).

Main suggestions :

1. As mentioned above, the manuscript at this stage does not provide a clear working model integrating the various results. This is particularly relevant when trying to integrate the various experiments perturbing MyoII in the skin or in the placode. Currently, the manuscript data suggest that :

i) Placode growth (through cell division and radial intercalation) is the main driver of orifice expansion

Yes, we agree with this statement.

ii) Orifice expansion is slow down/balanced by the MyoII purse string at the skin interface

We would like to clarify that the myosin II ring is not located in the skin cells but in the upper border of the cavity, meaning at the apical tips of olfactory placode cells (and indeed close to the interface with the overlying skin cells). To make it clearer in the article, we added several sentences stating this observation throughout the revised text.

Moreover, inhibiting tension within the ring through tissue-specific expression of dominant negative myosin II in olfactory placode ciliated neurons led to a slight reduction of the size of the orifice in the skin, and a modified -more wiggly- orifice shape, but no change in cavity area was observed (Fig. 4d, d', e, e', e''). These data suggest that the placodal actomyosin ring does not function as a purse string, as it does not seem to counteract orifice or cavity expansion but rather promotes the smoothening of the orifice edge. We modified a sentence in the discussion to clarify this point, page 13. Our results on ERK signaling suggest that the 3D cavity morphology (purse-like shape versus open shape) is rather regulated by actomyosin contractility in the skin (negatively: the reduction of skin contractility downstream of ERK promotes the opening of the cavity and the expansion of the orifice).

iii) Skin tension/MyoII mostly act as a limitant factor for placode planar expansion (but not necessarily its real surface, see my point on 3D measurement)

Yes, our data support the notion that actomyosin contractility in the skin acts as a limitant factor for the flattening/opening of the cavity, which is, from what we understand, what reviewer 2 means by placode planar extension.

iv) MyoII depletion in the placode perturb its expansion.

We observed different effects of myosin II inhibition on placode cavity area, depending on the experimental condition:

- A reduced cavity area was observed upon global treatment with ML7 and Rockout drugs (Fig. 4c, c') suggesting indeed that myosin II activity is important for cavity (or placode) growth, for instance for cell divisions in the basal region of the placode or for intercalation of new cells in the rosette.
- However, as precised above, inhibiting actomyosin activity specifically in olfactory placode ciliated neurons with the Gal4/UAS system did not affect cavity area (Fig. 4d, d', e, e', e''). Thus, in this condition, the perturbation of myosin II in the placode does not perturb cavity expansion. This may be due to the fact that most cells targeted by the *Tg(ompb:Gal4FF)* driver used in this experiment are already incorporated in the rosette (expression was barely detected in more basal regions of the placode), hence preventing any effect on cell divisions in the placode or cell intercalation behaviours. We added this information in the text page 9:

"To do so, we combined the *Tg(ompb:Gal4FF)* driver[43], which starts to be expressed at around 30 hpf in a subset of ciliated sensory neurons already incorporated in the rosette (thus preventing any effect on the opening, placodal cell division and cell intercalation in the rosette), with the *Tg(uas:DNmyosin-Scarlet)* line[17]."

In this framework, if the placode indeed push on the skin boundary and is constraiedn by the actomyosin pursestring, it should tend to be under pressure (which match the negative impact of laser ablation in the placode on final orifice size).
Could the authors use their ablation experiment in the placode (potentially ablating a large disc of cells) and check on short time scale whether this is associated with

expansion or contraction of the empty area ? This would provides valuable information about the tensional state of the placode.

According to our results and as explained above, the placode indeed pushes and exerts outward forces on the skin boundary (as it grows through progressive intercalation of new cells). It is not constrained by the placodal actomyosin ring (since the actomyosin ring does not act as a purse string), but rather by contractility in the skin, which is itself balanced/downregulated by the ERK pathway.
As suggested by the reviewer, we performed multicellular laser cuts within the placode tissue to assess its tensional state during orifice expansion. A slight recoil was observed after ablation, indicating that at these stages the placodal tissue may be under mild tension rather than compression. We present these new results in Supplementary Fig. 4f and in the text page 10:

"To further map mechanical stresses within the placode tissue we performed linear, supracellular laser cuts in the placode of *Tg(cldnb:meGFP)* embryos at various stages of orifice expansion. A subtle recoil was observed following the ablation, suggesting that at these stages the placode tissue may be slightly under tension, but not under compression (Supplementary Fig. 4f). This mild tension could be due to intrinsic forces generated in the placode or extrinsic forces exerted by nearby tissues (skin, or brain[72])."

2. As mentioned above, the authors mostly use z projection to measure the cavity area. However this may poorly reflect the total number of cells and real surface of the cavity/placode if there is significant bending. Could the author provide some estimate of the real 3D surface of the cavity in all the contexts ? This will help to decouple the parameters impacting growth of the placode versus the context impacting its capacity to laterally expand (which probably relies also on the relative stifness/tension between skin and placode). If the 3D measurement is too tedious with the resolution they have, they should at least provide some lateral view and an estimation of the number of cells in the cavity.

We thank the reviewer for this important remark. In the submitted article, we analysed 2D cavity areas on maximum projections, and the 3D shape of the cavity was assessed with the cavity shape ratio in the ERK perturbation experiments. We clarified the definition of the cavity shape ratio by improving the schematic view in Fig. 5b''' and giving the following definition in the Materials and Methods section (page 20): "The cavity shape ratio was calculated by dividing the area of the most apical part of the cavity by the area of the projected cavity."

As proposed by the reviewer, to better estimate the 3D cavity shape and 3D cavity surface, we used orthogonal sectioning along the ML and AP axes of the cavity and quantified several size/shape descriptors (cavity profile length, lateral aspect ratio and average curvature, as defined in Fig. 3d and Supplementary Fig. 3e). We could not perform this analysis in all the contexts, since it requires high-resolution images. We therefore chose to apply this 3D analysis protocol to the time-course experiment in the wild type situation, a condition for which we had already acquired high-resolution images (new analyses presented in Fig. 3d', d'' and

Supplementary Fig. 3e'), as well as to the new experiments in which we perturbed ERK signaling and fixed embryos at two stages, 40 and 48 hpf (analyses presented in Supplementary Fig. 6), since we had already spotted defects in 3D cavity shape upon ERK inhibition through the analysis of the cavity shape ratio.

We commented on these new results in the text page 7 (for the wild type time course):
"Qualitative observation of orifice and cavity orthogonal sections (Fig. 1c) suggests that the 3D cavity shape evolves during orifice expansion. To evaluate cavity shape and 3D surface, we used orthogonal sectioning along the ML and AP axes on high-resolution images and quantified several size/shape descriptors, as described in Fig. 3d and Supplementary Fig. 3e. As expected, the length of the cavity border on these sections (profile length) increased over time, both in the ML and AP dimension, reflecting the cell intercalation-mediated growth of the cavity surface in 3D (Fig. 3d', d''). Moreover, the initially pocket-like and curved morphology of the cavity border progressively transitioned into a flatter and more open shape during orifice expansion (Fig. 3d', d'' and Supplementary Fig. 3e'). These data show that a gradual opening and flattening of the cavity occurs alongside the increase in its three-dimensional surface area during orifice expansion."

And page 10 (for the new pharmacological ERK inhibition experiments):
"To further analyse when and how these phenotypes arise, we performed a time-course experiment in which we treated embryos from 32 hpf and fixed them at 40 hpf and 48 hpf. High resolution images were acquired to evaluate 3D cavity surface and shape, and quantify the number of small/round apical tips contributing to the cavity border. The cavity and orifice defects were already clearly visible at 40 hpf in PD184352-treated embryos (Supplementary Fig. 6a, b), indicating that ERK signaling is required from the early stages of expansion, consistent with our live imaging experiments. The data further confirmed that the smaller cavity 2D area observed upon ERK inhibition results from increased cavity curvature, combined with a slight (but not significant) reduction in its 3D surface area at 48 hpf and in the number of small, round apical tips (Supplementary Fig. 6c-e). Comparison of 3D cavity shape parameters between PD184352-treated embryos at 40 hpf and wild-type embryos at 32 hpf (Supplementary Fig. 6d, e, compare with Fig. 3d', d'') suggests that the cavity morphology phenotype reflects more than a simple developmental delay."

We also modified Figure 6 to visualise the 3D cavity shape changes (curved to flat transition) occurring over time in the wild type context.

3. Results in Figure 4d suggest that MyoII in the placode promotes orifice growth. Right now I see two ways to interpret this : either MyoII inhibition impact placode cell division and/or radial intercalation, or alternatively MyoII depletion affect the global stifness/bending modulus of the placode which cannot "push" anymore effectively on skin boundary (this is relevant only if the placode is indeed under pressure). Have the authors any way to estimate the number of placode cells in this context (inhibition of MyoII in the placode versus control) ? The fact that placode area remain the same (provided there is no change of bending) may suggest that division/intercalation are not

significantly affected and would suggest that it is mostly the capacity to "push" that is limited.

Please see the response to reviewer 2's point 1, statement iv).

In addition, as suggested by the reviewer we quantified the number of small and round apical tips in *Tg(ompb:Gal4FF); Tg(uas:DNmyosin-Scarlet)* embryos for which high-resolution confocal images were available, as well as the area of individual tips. The results, which confirm that the number and area of small and round apical tips is not significantly changed in the cavity of double transgenic embryos, are integrated in Supplementary Fig. 4e, e'. This reinforces the idea that placode cell divisions and intercalation events are not affected in this condition.

4. The results on ERK are interesting but seems a bit preliminary to build a firm model. If the authors want to claim that ERK inhibits tension in the skin, they should then either compare the levels of actomyosin in DMSO and MEK inhibited skin and/or perform laser ablation in the skin to compare tension.

We went back to our images and did not detect major qualitative changes in the levels of myosin II and F-actin in skin cells upon ERK inhibition, using the *Tg(actß2:myl12.1-GFP)* line and phalloidin staining, respectively. However, our functional data -the rescue of the cavity shape defect by the downregulation of myosin II activity specifically in the skin- strongly argue that ERK inhibition increases tension/contractility in the skin (Fig. 5d, d').
Additional observations further support the hypothesis of a role for a dampening of skin contractility downstream of ERK signaling. For instance, the mean border length of the bordering cells is strongly reduced at 48 hpf in PD184352-treated embryos, and live imaging confirms that the orifice first neighbors shorten their border length over time, at least during the first 6 hours of treatment (Fig. 5c, c'), leading to a progressive closure/collapse of the orifice. We added a sentence page 10 stating that we observed a reduction of the border length in the live imaging data. These cell shape changes are additional indicators of a higher contractility of the skin cells upon PD184352 treatment, at least along the orifice edge. Last, with our new experiments using the *Tg(krt4:KalTA4)* driver to inhibit actomyosin contractility in the skin (Supplementary Fig. 5d), we have further proof that skin actomyosin contractility also counteracts the opening of the orifice/cavity structure in the wild type situation.

5. Similarly, do they have some estimation of the impact of ERK inhibition on placode growth/radial intercalation (as they did in the skin) ? Measuring real 3D surface of the placode would be quite essential here.

To address this point, we first quantified the number of small, round apical tips in our previous ERK inhibition dataset at 48 hpf (when high-resolution images were available), as well as at 40 and 48 hpf in our new ERK inhibition experiments. In the original dataset, we observed a significant reduction in the number of small, round apical tips in PD184352-treated embryos, suggesting that ERK signaling contributes to placode cell divisions and/or cell intercalation within the rosette. These results are presented in Supplementary Fig. 5c and discussed page

11. A similar trend was observed in the new ERK inhibition experiments at both 40 and 48 hpf; however, this did not reach statistical significance using ANOVA with multiple comparisons (Supplementary Fig. 6c). This consistency nevertheless supports our initial conclusion. In addition, in the new experiments we used the anteroposterior (AP) and mediolateral (ML) profile lengths of the cavity as proxies for its 3D surface. Although no statistically significant differences were detected, a trend towards reduced profile length was apparent at 48 hpf in PD184352-treated embryos. Taken together with the clear impact of ERK inhibition on 3D cavity morphology (increased curvature/bending), these findings suggest that the reduced 2D cavity area initially observed in PD184352-treated embryos results from a combination of decreased rosette cell number and enhanced 3D constriction or folding of the cavity. These points are addressed on page 11.

6. The authors do not really comment the phenotype of the orifice upon perturbation of MyoII in the skin (Figure 5d,d', comparing DMSO and DMSO krt5 DNMyoII). While their results on ERK and tissue bending suggest that skin tension can prevent the expansion of the orifice due to hypercontractility, it does not really fit with the absence of orifice area increase upon MyoII inhibition in the skin. Would the authors have some hypothesis to explain this ?

See our response to reviewer 1, point 4.

7. Along this line, intuitively skin tension may have opposing contribution to the expansion of the cavity, with bulk tension promoting expansion (provided that skin is indeed under tension) and the pursestring rather slowing down the expansion of the orifice. This may explain the surprisingly poor impact of MyoII inhibition in the skin on orifice area. Could the authors provide some speculation along this line ? (I suspect there is no way experimentally to specially block specifically MyoII in the purse string versus the bulk of the skin).

As clarified above (reviewer 2, point 1), the myosin II ring is not found in the skin but rather in the upper edge of the cavity, within the apical tips of olfactory placode neurons, near the interface with the overlying skin cells.
Overall our data suggest that bulk tension in the skin has a negative effect on orifice growth. See also reviewer 1 point 4 response where we describe the additional experiments we made with the new *Tg(krt4:KalTA4)* line to drive the expression of the dominant negative myosin II in the skin. In this condition we observed an increased orifice and cavity areas at 48 hpf, which, together with our ERK results, support the notion that actomyosin contractility/tension in the skin counteracts the growth of the olfactory orifice and exerts a retroaction on the cavity.

8. The authors characterize in figure 3 the expansion of the cavity through the number of small apical round area and the later inclusion of large cuboidal cells. However the surface in between the small apical area may also change and contribute to area expansion. Could the authors document also the evolution of spacing and/or density of the small apical area cells during development ? That would provide an exhaustive

characterization of what contribute to placode growth. Moreover, could they provide some information on what stand in between those cells (other epithelial cells ? ECM ? mesenchymal cells ?). ?

To address this point we measured the total surface covered by these ZO-1-negative regions and showed that it increases over time (and their proportion in the cavity area remains the same over time), indicating that these regions contribute to cavity growth (Supplementary Fig. 3a). To assess whether these regions are acellular spaces or contain cellular material, we injected GFP mRNA at one-cell stage, which leads to ubiquitous GFP expression, into *KI(zo1a:zo1a-tomato)* embryos (reference 67) to label the ZO-1- positive apical surfaces lining the cavity. Most of the ZO-1-negative area exhibited GFP signal (Supplementary Fig. 3a'), indicating that it represents cells that contribute to the cavity border but do not express ZO-1 apically. We commented on these new observations, page 6 (see below), showed a representative image in Supplementary Fig. 3a', and modified our conclusion page 6 and our schematics in Figures 3b and 6.

"In addition, we noticed the presence of areas devoided of ZO-1 staining intermingled with the small and round ZO-1-positive apical tips. The total surface covered by the ZO-1-negative regions increased during orifice expansion (and their proportion remained constant), indicating that they contribute to cavity growth (Supplementary Fig. 3a). ZO-1-negative regions mainly corresponded to cells that do not seem to express ZO-1 apically, and occasionally to small acellular spaces, as observed in embryos ubiquitously expressing cytoplasmic GFP (Supplementary Fig. 3a')."

Other minor points :

1. It is interesting to note that the recoil speed of the skin interface (Fig. 4b) does not seem to change with cavity area. It might be worth mentioning this point in the main text/discussion.

In Fig. 4b the recoil speed following the ring ablation is not that of the skin interface but that of the non-ablated portions of the ring located in the upper part of the placodal cavity. We chose to represent the recoil speed as a function of cavity area to illustrate that we analysed several developmental stages covering the initial expansion phase, but in our opinion the number of samples analysed at various stages does not allow to conclude firmly about the presence or absence of tension evolution over time.

2. Could the authors provide measurement of the cavity perimeter and its smoothness in all the context perturbing MyoII (Figure 4) ? Right now, only the area is provided, but I suspect that perimeter is mostly unaffected (since number of bordering cells and border lengths are maintained). This would fit with a more irregular perimeter as well (hence increase perimeter other area ratio).

As suggested by the reviewer, we measured cavity perimeters and solidities in embryos treated with ML7 and Rockout and in *Tg(ompb:Gal4FF); Tg(uas:DNmyosin-Scarlet)* embryos. While

Tg(ompb:Gal4FF); Tg(uas:DNmyosin-Scarlet) embryos display no significant changes in cavity perimeter and solidity, ML7+Rockout treatment results in a reduction of both parameters. The decreased solidity could be due to changes in how cells organise at the cavity border upon global myosin II inhibition. These results are shown in Supplementary Fig.4c, c', d, d'.

3. Regarding the contribution of cell division in the skin, while I do agree that the data of the authors suggest that cell division is not absolutely necessary for orifice growth, I note however that the initial cell geometry and apical area is very much different in the CDKN1B context. One cannot exclude that the mechanical state of the system from the start is quite different. The ideal experiment would be to block cell division temporally specifically at the time of orifice growth, but that is not possible with the skin specific genetic inhibition. I may suggest to mention this limitation somewhere the text.

We agree with the reviewer. To address this point, we carried out new experiments to block skin cell divisions specifically during orifice expansion using a homemade, inducible Gal4 line, *Tg(krt4:Gal4-ERT2-VP16),* allowing an induction of mosaic expression in the skin from 30 hpf onwards. This had again no effect on orifice and cavity areas at 48 hpf, confirming that skin cell divisions are not required. Of note, this was impossible to achieve with the other inducible line used in this study, *Tg(krt5:Gal4-ERT),* since tamoxifen treatment at 24 hpf did not trigger any UAS transgene expression in our hands.

We presented these new results in Supplementary Fig.1d, d' and described them page 5:

"Next, to restrict the inhibition of skin cell division to the initial expansion phase rather than throughout development, we generated an inducible *Tg(krt4:Gal4-ERT)* line and combined it with *Tg(uas:cdkn1b-E2A-GFP)*. Tamoxifen induction at 24 hpf led to mosaic expression from 30 hpf onwards. No significant difference in orifice or cavity area was found at 48 hpf in double transgenic, induced embryos compared to siblings, and the orifice still grew through the deformation of bordering cells (Supplementary Fig.1d, d')."

Reviewer #3

Clara et al. reported a study on the dynamic evolution of the olfactory orifice and the placodal cavity in zebrafish. They investigated how the size of these epithelial holes evolve over time and the regulating roles of cell contractility and ERK signaling. This research is in-depth, systematic and interesting, bridging a critical gap in existing literature of tissue hole dynamics. The manuscript is well written with clear logic and sufficient evidence. My concerns are provided below.

1. The placodal cavity, i.e., the apical side of placodal rosette cell, exhibited a dome-like shape in the three-dimensional space. However, the authors quantified only the growth dynamics of the projected area. In my opinion, it would be interesting to examine how the curvature of such a dome-like cavity evolves over time. Does it become more curved or

flat as the cavity grows? In addition, it would also be interesting to quantify the shape of holes in the projected plane. I think these additional quantifications will strengthen this work.

For the analysis of the 3D shape/curvature of the cavity over time in the wild type context, see our detailed response to reviewer 2 point 2. This new analysis revealed that the cavity undergoes a transition from a curved/bended morphology towards a flat and open shape.
In addition, we quantified the solidity of the orifice in our time course experiments on fixed embryos (what we think the reviewer meant by *shape of holes in the projected plane*). No change in orifice solidity was detected over time, we presented these data in Supplementary Fig. 1c and described them with a new sentence, page 4.

2. The authors quantified the dynamic evolution of olfactory orifice and placodal cavity starting from 32 hpf with the orifice area ~100 um2 and cavity area ~ 200 um2, much larger than zero. Why there is no data before 32 hpf? (Small holes difficult to detect?) More discussion is required.

We previously published a study analysing the mechanisms driving the initial opening of the olfactory orifice which occurs around 32 hpf (Baraban, M. *et al.* Actomyosin contractility in olfactory placode neurons opens the skin epithelium to form the zebrafish nostril. *Dev. Cell* **58**, 361-375.e5 (2023). In the present work, we chose to focus specifically on the subsequent expansion stage, as outlined in the introduction and the first section of the results.

3. The clarity of the measurement method of the cavity area should be improved.

The method we used to measure the cavity area in 2D, on maximum projections, is described in the Material and Methods sections, page 19:
"ZO-1 immunostaining or phalloidin staining were used to analyze the area of the placodal cavity through the freehand selection tool and ROI manager plugin of ImageJ, on 2D maximal projections. This step was performed in a blind manner, meaning without visualizing the overlying orifice in the skin."

In addition, we now provide a deep analysis of the cavity size and shape in the third (z) dimension in the wild type time course experiment (Fig. 3d, d', d'') and in the ERK inhibition section (Supplementary Fig. 6d, e).

4. If the first or second neighbors are damaged, how will the tissue carry out self-rescue? What changes will occur in cell movement or morphogenesis?

We performed the suggested ablation of individual bordering skin cells at 32 hpf and assessed how the system responds to this perturbation by monitoring skin cell behaviours during orifice expansion. We presented the data in the new Supplementary Fig. 2 and concluded the following in the text (page 4):

“To further characterise bordering skin cell behaviours, we ablated individual bordering cells at 32 hpf and observed how the system reacts. The ablation triggered rapid remodeling of adjacent skin cells and immediate contraction of the orifice (Supplementary Fig. 2a, b, b’), resembling a skin wound-healing response[20,21]. Over the longer term however, the impact on orifice expansion was minimal, since orifice area and number of bordering cells remained comparable to those in unablated 48 hpf embryos (Fig. 2b, b’’ and Supplementary Fig. 2b’’, b’’’), showing that the growth process is robust to bordering cell damage.”

5. Why the shape of the dorsal view is oval and has the characteristics of long axis and short axis? Is it related to the distribution of different types of cells? Or it is related to the distribution of actomyosin?

As spotted by the reviewer, the cavity is oval, with its AP dimension being higher that the ML dimension. In a previous study we showed that the cavity already has this oval shape during the initial opening of the orifice. At these early stages the elongated cavity morphology correlates with the presence of anterior and posterior actomyosin cables in rosette cells. We showed that these cables are under tension and proposed that they pull on and deform the cavity border (see reference 17). However during the expansion phase it is more difficult to relate the oval cavity shape to actomyosin distribution (absence of radial cables in placode cells for instance) or cell type distribution. The precise mechanism that allows the maintenance of this oval shape during the expansion phase will be analysed in future studies.

6. Just from the results of the cross-sectional diagrams (Fig. 1a), there are differences in the shapes of the cavity in the early (32-36 hpf) and late (48 hpf) stages. After interfering ERK pathway, the shape of the cavity is similar to that in the early stage (32-36 hpf). Does the effect of ERK pathway have a stage-specific functions? Does the ERK pathway have a minor effect in the initial expansion stage and a more significant effect in the later expansion stage?

To address these questions we have generated a new set of data to analyse the effect of ERK inhibition at an intermediate stage (40 hpf). We present these new results in Supplementary Fig. 6 and comment on them pages 10-11:

“To further analyse when and how these phenotypes arise, we performed a time-course experiment in which we treated embryos from 32 hpf and fixed them at 40 hpf and 48 hpf. High resolution images were acquired to evaluate 3D cavity surface and shape, and quantify the number of small/round apical tips contributing to the cavity border. The cavity and orifice defects were already clearly visible at 40 hpf in PD184352-treated embryos (Supplementary Fig. 6a, b), indicating that ERK signaling is required from the early stages of expansion, consistent with our live imaging experiments. The data further confirmed that the smaller cavity 2D area observed upon ERK inhibition results from increased cavity curvature, combined with a slight (but not significant) reduction in its 3D surface area at 48 hpf and in the number of small, round apical tips (Supplementary Fig. 6c-e). Comparison of 3D cavity shape parameters between PD184352-treated embryos at 40 hpf and wild-type embryos at 32 hpf (Supplementary Fig. 6d,

e, compare with Fig. 3d’, d’’) suggests that the cavity morphology phenotype reflects more than a simple developmental delay.”

*note about the replacements of panels in the figures:
We have replaced the images in panel Fig. 1c and the circular plots in panel Fig. 2c’’’. These changes do not affect our previous conclusions.
