# Supplementary material for: Dynamic interactions between epithelial skin cells and a sensory cavity sculpt the growing olfactory orifice
Source: Nat Commun. 2026 Jul 8;17:8452. doi: 10.1038/s41467-026-75307-y (PMC13478634; doi:10.1038/s41467-026-75307-y)
Supplement: Supplementary file 3 [file 41467_2026_75307_MOESM1_ESM.pdf]

Supplementary Figure 1 Gordillo Pi et al.

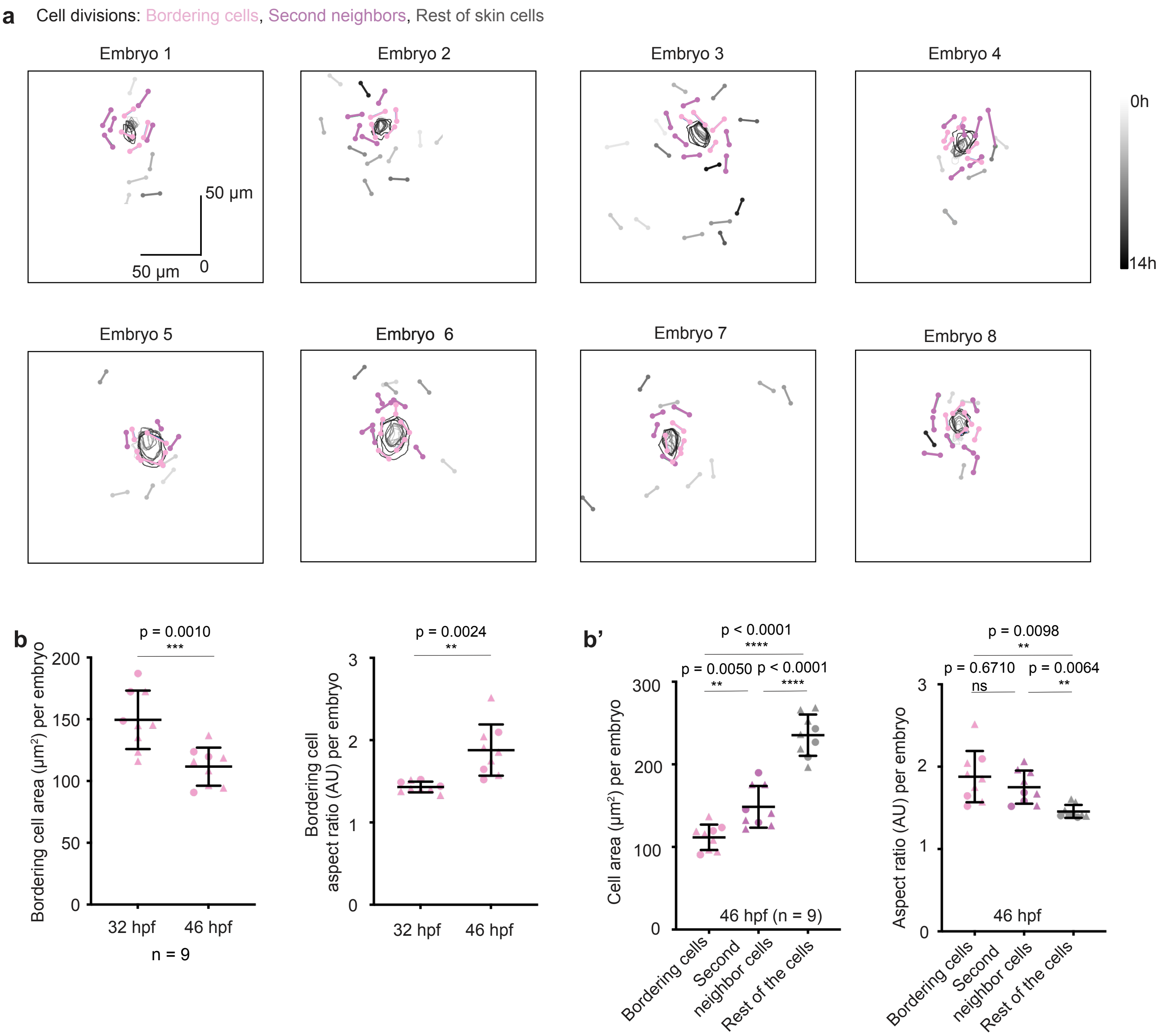

Supplementary Figure 1. Additional data on skin cell remodeling during orifice expansion, related to Fig. 1 and Fig. 2**. a.** Maps of cell divisions occurring during the time lapse sequence in the field of view, related to Fig. 2c'. Grey lines = contours of the growing orifice, the time is color coded. For each division, a segment links the centers of the two daughter cells, which are represented by the dots. Pink segments = 1st neighbor skin cell divisions, purple segments = 2nd neighbor cell divisions, grey segments = divisions in the rest of the skin epithelium (for those, time is color coded). **b.** Comparison of the area or aspect ratio of bordering cells at 32 and 46 hpf (n = 9 embryos from 2 independent experiments). Unpaired, two-tailed t-test for the area, and Welch's t-test for the aspect ratio. **b'.** Comparison of the area or aspect ratio of bordering cells, second neighbor cells and rest of the skin cells at 46 hpf (9 embryos from 2 independent experiments). ANOVA followed by Tukey multiple comparison test for the cell area, Welch's ANOVA followed by Dunnett T3 multiple comparison test for the aspect ratio.

Supplementary Figure 2 Gordillo Pi et al.

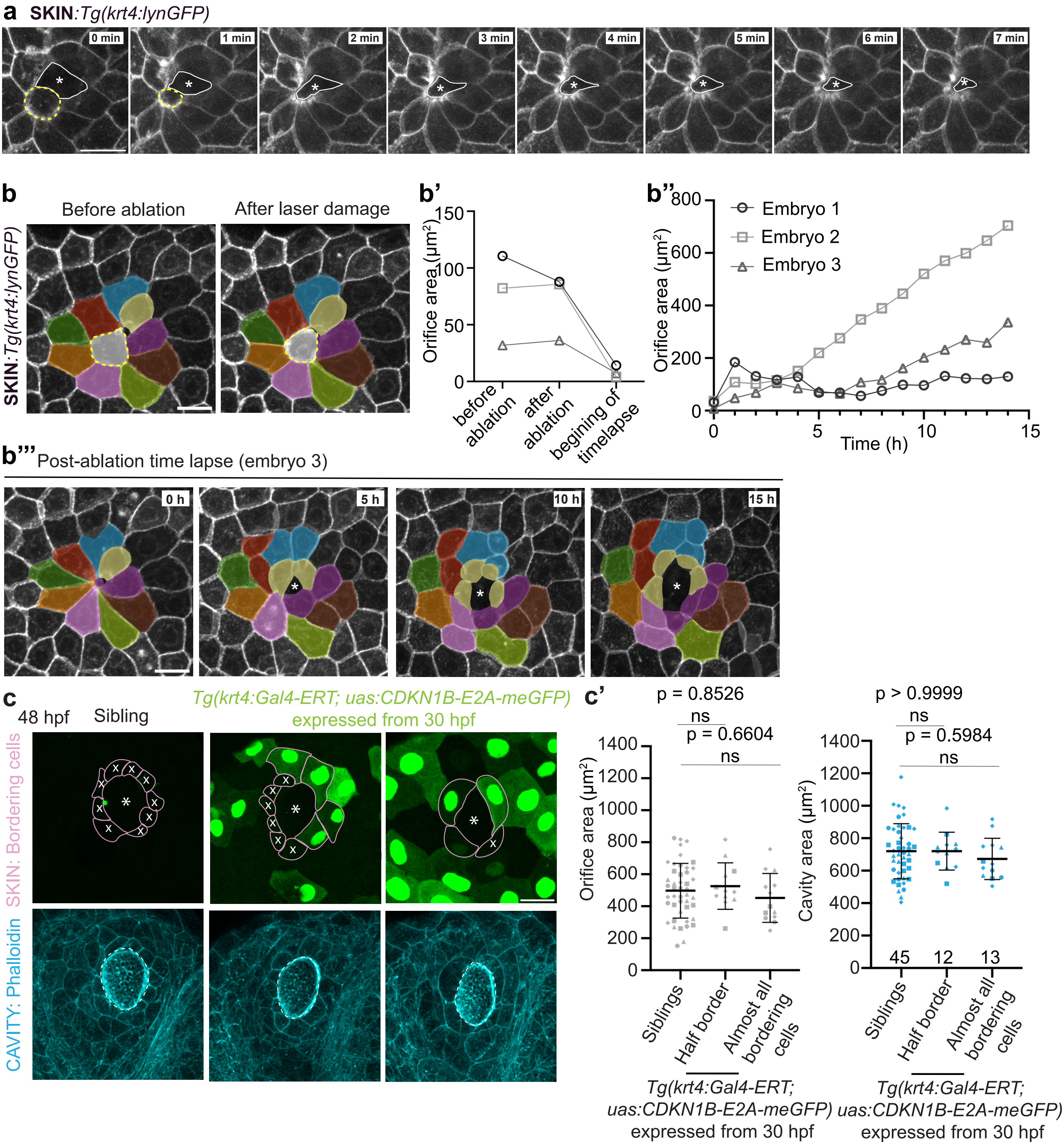

Supplementary Figure 2. Skin remodeling following bordering cell ablation, related to Fig. 2**. a.** Live imaging on a *Tg(krt4:lynGFP)* embryo during the first 7 minutes following the laser damage of a bordering cell. Dorsal view, anterior on the top, lateral on the left, maximum projections. Yellow dotted line = laser damage cell. White line = orifice contour. Asterisk = orifice. Scale bar = 20 µm. **b.** Images of another *Tg(krt4:lynGFP)* embryo (embryo 3) before and after laser damage. Cells located on the orifice edge as well as cells in contact with the laser damage cell (white) are colored. Yellow asterisk = ablated cell. Scale bar = 20 µm. **b', b''.** Quantification of orifice area before and after laser damage, and at the beginning of the time lapse (b'), and during the 15 hours following laser damage (b'') (n = 3 movies from 2 independent experiments). **b'''.** Live imaging of 15 hours post-laser damage (embryo 3). Scale bar = 20 µm. **c.** Images of *Tg(krt4:Gal4-ERT; uas:cdkn1b-E2A-GFP)* embryos, in which cell divisions are inhibited in the skin during the initial expansion phase (GFP expression arises from 30 hpf, after tamoxifen induction at 24 hpf), and control siblings fixed at 48 hpf, stained with phalloidin-rhodamine (cyan), GFP in green. Top images = Maximal projection of the GFP channel. Asterisk = orifice. Bordering cells are outlined with a pink line. Crosses indicate bordering cells which do not express GFP. Bottom images = cavity seen with phalloidin-rhodamine staining, maximum projections. Dotted white line = cavity contour. Scale bar = 20 µm. **c'**. Quantification of orifice area, cavity area in *Tg(krt4:Gal4-ERT; uas:cdkn1b-E2A-GFP)* embryos and control siblings at 48 hpf (4 independent experiments, n = 25 double transgenic embryos (12 with half of the bordering cells expressing GFP, and 13 with almost all bordering cells expressing GFP), n = 45 control siblings). Tukey's multiple comparisons test.

Supplementary Figure 3 Gordillo Pi et al.

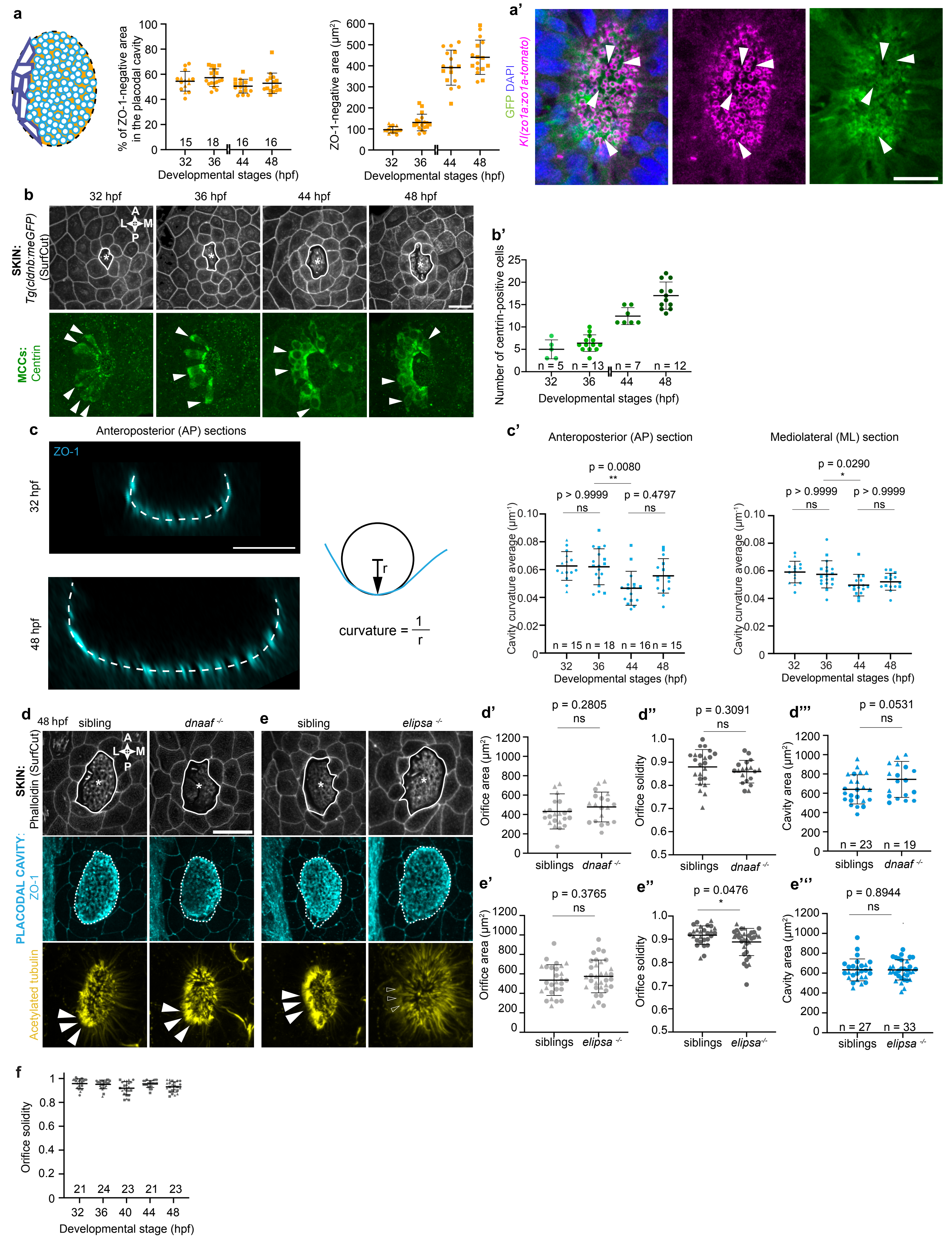

Supplementary Figure 3. Additional data on the cavity, ~~on~~ MCCs and analysis of ciliary mutants, related to Fig. 3 and Fig. 1**. a.** Schematic dorsal view of the placodal cavity at 48 hpf, with small apical tips of olfactory sensory neurons (light blue), large apical tips of MCCs (dark blue) and ZO-1 negative areas (orange). Quantification of the total area covered by ZO-1 negative regions over time, and of the percentage of the cavity area it corresponds to. Related to Fig. 3c-3d''. **a'.** Images of a *TgKI(zo1a:zo1a-tomato)* embryo injected with GFP mRNA and immunostained for GFP, Tomato, and labelled with DAPI. The arrow points to an empty space, likely acellular, while the arrowheads indicate ZO1-negative cell surfaces. Scale bar = 10 µm **b.** Images of *Tg(cldnb:meGFP)* embryos immunostained for Centrin, time course analysis. Top panels show *Tg(cldnb:meGFP)* expression in the skin (grey) extracted with the SurfCut plugin, bottom panels show Centrin expression (green, 1 z-section). Arrowheads point to Centrin-positive MCCs on the lateral side of the cavity. Dorsal view. Asterisk = orifice. White line = orifice contour. Scale bar: 20 µm. **b'.** Number of Centrine-positive cells, time course analysis (1 experiment, 32 hpf: n = 5 embryos; 36 hpf: n = 13 embryos; 44 hpf: n = 7 embryos; 48 hpf: n = 12 embryos). **b.** Images of *dnaaf* $^{-/-}$ mutant embryos and control siblings at 48 hpf. Top images = skin labelled with phalloidin (grey) and extracted with the SurfCut plugin, middle images = cavities seen with ZO-1 immunostaining (maximum projections), bottom images = Acetylated-tubulin immunostaining to reveal the cilia (more generally acetylated microtubules). Asterisk = orifice. White line = orifice contour. Dotted white line = cavity contour. Arrowheads indicate the multiple cilia of MCCs in the lateral region of the cavity, present in both controls and *dnaaf* $^{-/-}$ mutants. Scale bar = 20 µm. **c.** High resolution image of ZO-1 immunostaining (orthogonal view of the AP section) at 32 hpf (top) and 48 hpf (bottom) and definition of the curvature in a given point of the cavity profile. Scale bar = 10 µm. **c'.** Quantification of the cavity curvature average on AP and ML sections (2 independent experiments, 32 hpf: n = 15 embryos; 36 hpf: n = 18 embryos; 44 hpf: n = 16 embryos; 48 hpf: n = 15 embryos). ANOVA followed by Kruskal-Wallis multicomparison tests. **d.** Images of *dnaaf* $^{-/-}$ mutant embryos and control siblings at 48 hpf. Top images = skin labelled with phalloidin (grey) and extracted with the SurfCut plugin, middle images = cavities seen with ZO-1 immunostaining (maximum projections), bottom images = Acetylated-tubulin immunostaining to reveal the cilia (more generally acetylated microtubules). Asterisk = orifice. White line = orifice contour. Dotted white line = cavity contour. Arrowheads indicate the multiple cilia of MCCs in the lateral region of the cavity, present in both control siblings and dnaaf$^{-/-}$ mutants. Scale bar = 20 µm. **d', d'', d'''**. Quantification of orifice area (d'), orifice solidity (d'') and cavity area (d''') in *dnaaf* $^{-/-}$ mutants and control siblings at 48 hpf (3 independent experiments, n = 19 *dnaaf* $^{-/-}$ mutants, n = 23 control siblings). Mann-Whitney test for orifice area, and unpaired, two-tailed t-test for cavity area. **e.** Images of *elipsa* $^{-/-}$ mutant embryos and control siblings at 48 hpf. Top images = skin labelled with phalloidin (grey) and extracted with the SurfCut plugin, middle images = cavities

seen with ZO-1 immunostaining (maximum projections), bottom images = Acetylated-tubulin immunostaining to reveal the cilia (more generally acetylated microtubules). Asterisk = orifice. White line = orifice contour. Dotted white line = cavity contour. Arrowheads indicate the multiple cilia of MCCs in the lateral region of the cavity, present in control siblings but not in *elipsa* $^{-/-}$ mutants. Scale bar = 20 µm. **e', e'', e'''**. Quantification of orifice area (e'), orifice solidity (e'') and cavity area (e''') in *elipsa* $^{-/-}$ mutants and control siblings at 48 hpf (2 independent experiments, n = 33 *elipsa* $^{-/-}$ mutants, n = 27 control siblings). Unpaired, two-tailed t-test for orifice area, and Mann-Whitney test for cavity area. **f.** Related to Fig. 1. Quantification of orifice solidity in fixed samples, according to developmental stages: 32 hpf: n = 21 embryos; 36 hpf: n = 24 embryos; 40 hpf: n = 23 embryos; 44 hpf: n = 21 embryos; 48 hpf: n = 23 embryos, related to Fig. 1b-f. ANOVA (p = 0.0256) followed by Kruskal-Wallis multicomparison test (not significant).

Supplementary Figure 4 Gordillo Pi et al.

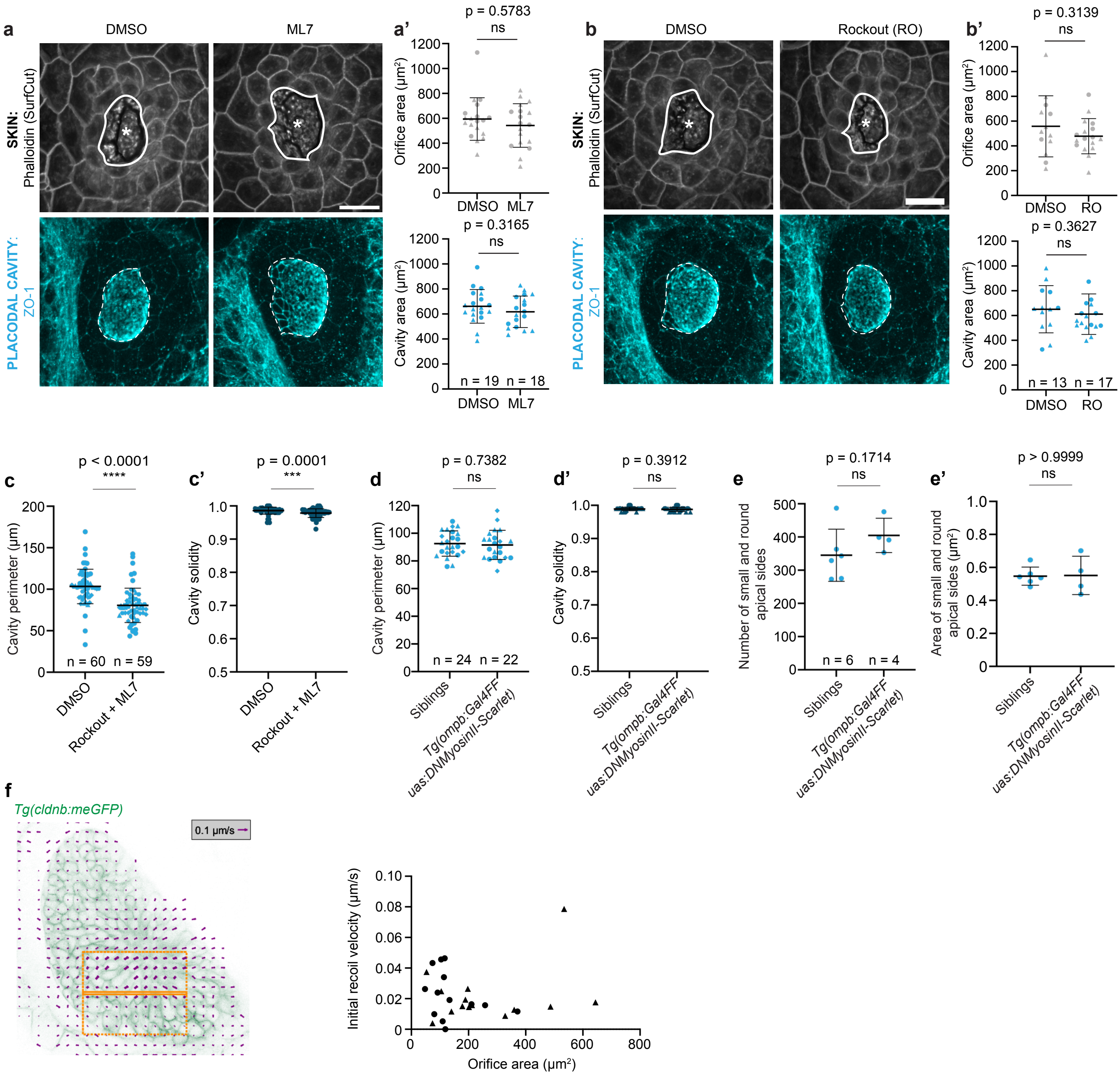

Supplementary Figure 4. Additional data on the inhibition of myosin II activity in the placode, and on placode tissue tension, related to Fig. 4**. a.** Images of 48 hpf embryos treated with ML7 or DMSO from 32 hpf. Top images = skin labelled with phalloidin and extracted with the SurfCut plugin (see Methods). Asterisk = orifice. White line = orifice contour. Bottom images = cavity seen with ZO-1 immunostaining, maximum projections. Dotted white line = cavity contour. Scale bar = 20 µm. **a'**. Quantification of orifice and cavity area in ML7-treated embryos and DMSO-treated controls at 48 hpf (2 independent experiments, n = 18 ML7-treated embryos, n = 19 DMSO-treated controls). Mann Whitney test for orifice area and unpaired two-tailed t-test for cavity area. **b.** Images of 48 hpf embryos treated with Rockout or DMSO from 32 hpf. Top images = skin labelled with phalloidin and extracted with the SurfCut plugin (see Methods). Asterisk = orifice. White line = orifice contour. Bottom images = cavity seen with ZO-1 immunostaining, maximum projections. Dotted white line = cavity contour. Scale bar = 20 µm. **b'**. Quantification of orifice and cavity area in Rockout-treated embryos and DMSO-treated controls at 48 hpf (2 independent experiments, n = 17 Rockout-treated embryos, n = 13 DMSO-treated controls). Welch's t-test for orifice area and Mann Whitney test for cavity area. **c, c'.** Quantification of cavity perimeter (c) and cavity solidity (c') in 48 hpf embryos treated with Rockout + ML7 or DMSO from 32 hpf (4 independent experiments, n = 59 Rockout and ML7-treated embryos, n = 60 DMSO controls). Related to Fig. 4c. Mann-Whitney tests. **d, d'.** Quantification of cavity perimeter (d) and cavity solidity (d') at 48 hpf in *Tg(ompb:Gal4FF; uas:DNmyosin-Scarlet*) double transgenic embryos or siblings (4 independent experiments, n = 22 double transgenic embryos, n = 24 controls). Related to Fig. 4d-e''. Welch's test for Supplementary 4d', Mann-Whitney tests for the rest. **e, e'.** Quantification of the number of small, circular apical tips and of their average area on high resolution images (1 experiment, n = 4 *Tg(ompb:Gal4FF; uas:DNmyosin-Scarlet*) double transgenic embryos, n = 6 siblings). Related to Fig. 4d-e''. Mann-Whitney tests. **f.** Supracellular linear laser cuts to probe tension in the placode. Example of a laser cut performed in the placode of a 40 hpf *Tg(cldnb:meGFP)* embryo (green). The image shows the embryo after the ablation (5s), with tissue velocity field overimposed (purple arrows) in two 20 µm-thick rectangles on each side of the ablation line. Dorsal view, 1 z-section. Scale bar = 10 µm. The graph shows the initial recoil velocity, used as a proxy for mechanical stress before the cut, as a function of the orifice area (n = 27 ablations, data pooled from 2 independent experiments).

Supplementary Figure 5 Gordillo Pi et al.

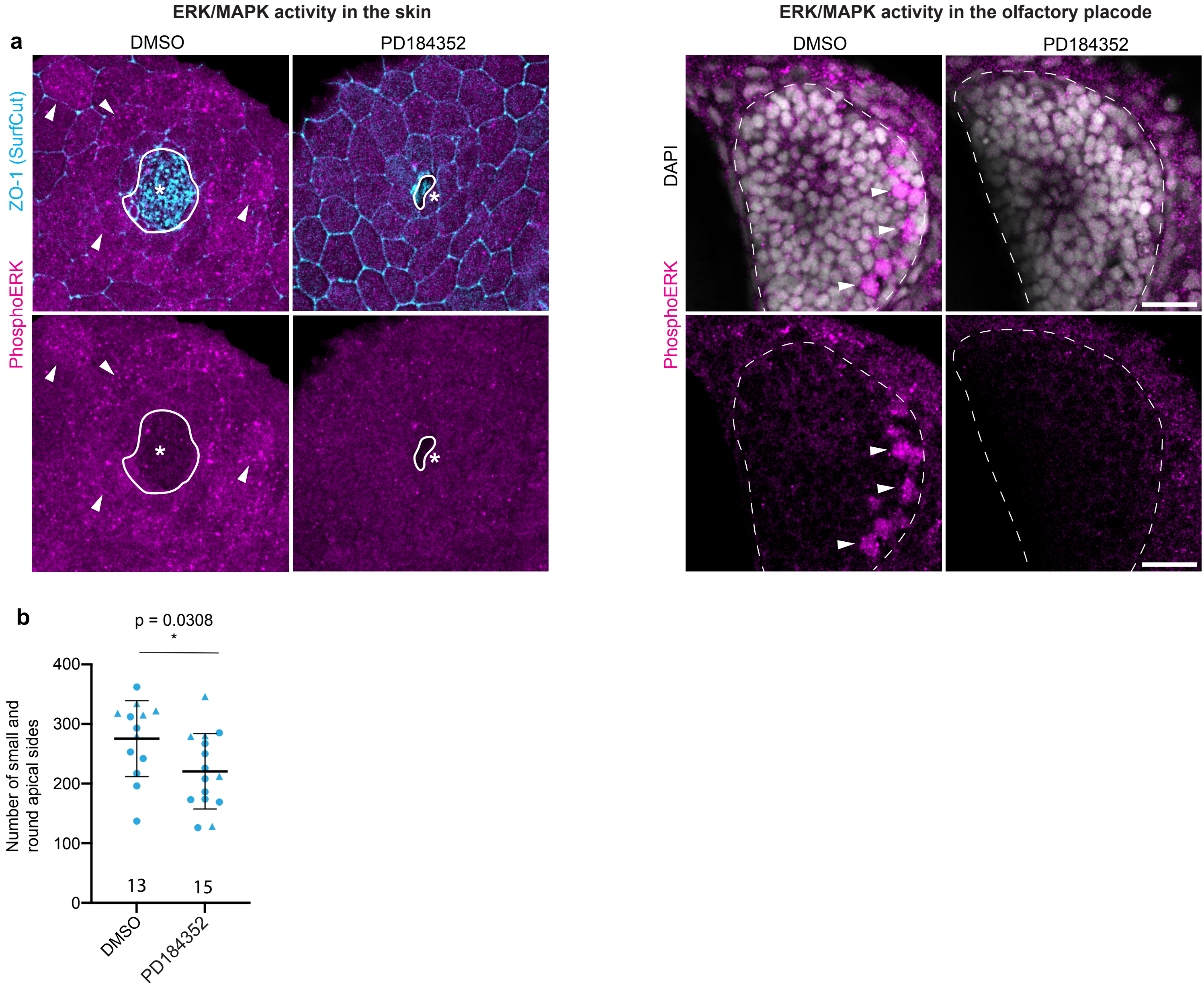

Supplementary Figure 5. Additional data on the pharmacological inhibition of ERK signaling, related to Fig. 5**. a.** Left. ZO-1 (cyan) and phospho-ERK (magenta) immunostaining in the skin, extracted with the SurfCut plugin (see Methods), in 48 hpf embryos treated with PD184352 or DMSO from 32 hpf. Arrowheads point to skin cells showing Phospho-ERK immunoreactivity in DMSO-treated controls. This staining is lost in PD184352-treated embryos. Asterisk = orifice. White line = orifice contour. Scale bar: 20 μm. Right. Phospho-ERK immunostaining (magenta) and DAPI staining (grey) in the olfactory placode in 48 hpf embryos treated with PD184352 or DMSO from 32 hpf. Dotted line = placode contour. Arrowheads point to olfactory placode cells showing Phospho-ERK immunoreactivity in DMSO-treated controls. This staining is lost in PD184352-treated embryos. Scale bar: 20 μm. **b.** Quantification of the number of small, circular apical tips lining the cavity at 48 hpf (2 independent experiment, DMSO treated: n = 13 embryos, PD184352 treated: n = 15 embryos). Unpaired, two-tailed t-test.

Supplementary Figure 6 Gordillo Pi et al.

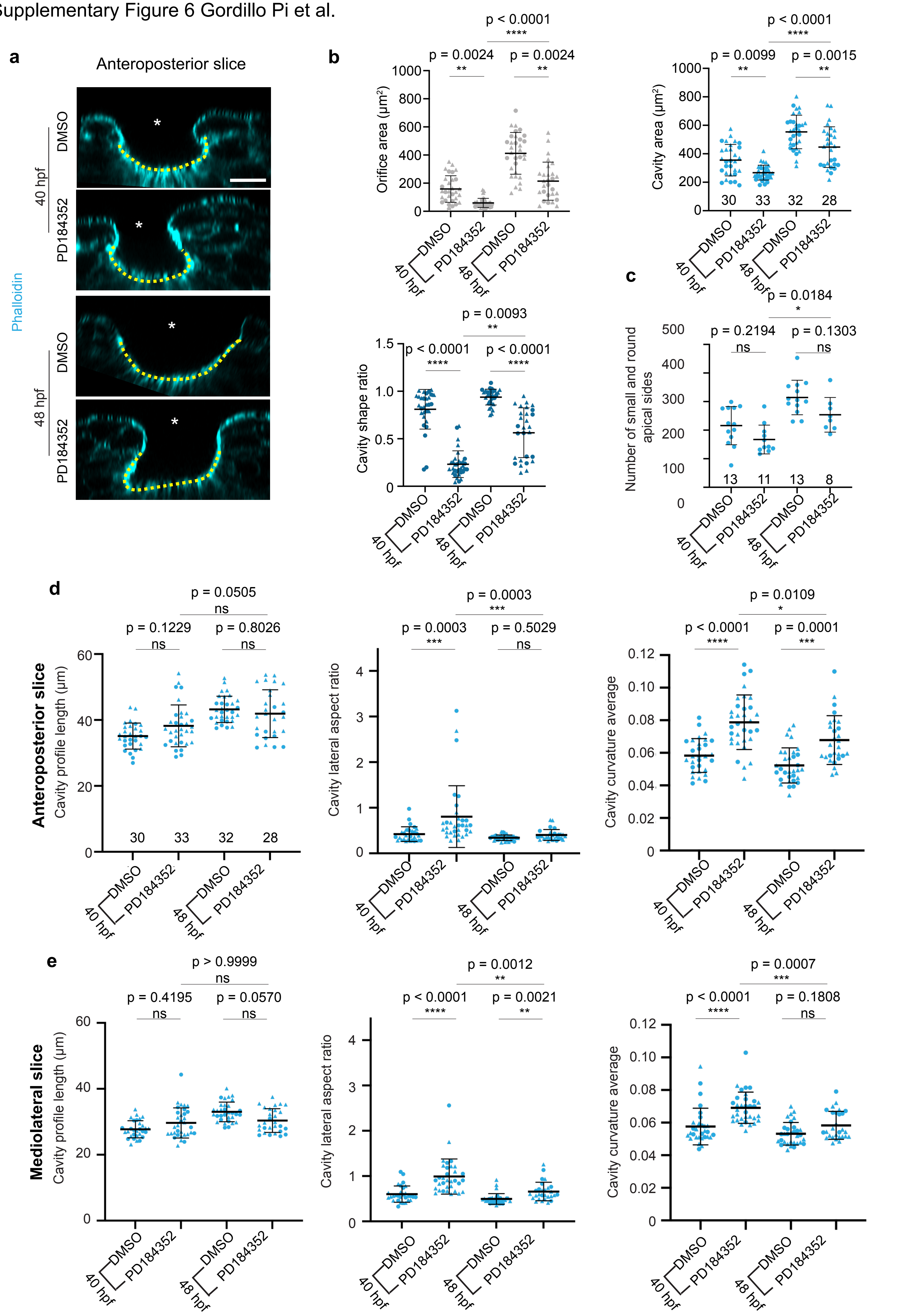

Supplementary Figure 6. Additional data on the pharmacological inhibition of ERK signaling - time course experiment, related to Fig. 5**. a**. Images of 40 hpf and 48 hpf embryos treated with PD184352 or DMSO from 32 hpf. Orthogonal AP sections of embryos stained with phalloidin-rhodamine (cyan) to reveal the cavity. Yellow dotted line = cavity profile. Asterisk = orifice. Scale bar = 10 µm. **b**. Quantification of orifice area, cavity area (on maximum projections), and cavity shape ratio upon PD184352 treatment (2 independent experiments, DMSO treated: n = 30 embryos at 40 hpf, PD184352 treated: n = 33 embryos at 40 hpf, DMSO treated: n = 32 embryos at 48 hpf, PD184352 treated: n = 28 embryos at 48 hpf). ANOVA followed by Tukey multiple comparison test for cavity area; ANOVA followed by Kruskal-Wallis multicomparison test for the two other graphs. **c.** Quantification of the number of small, circular apical tips lining the cavity (1 independent experiment, DMSO treated: n = 13 embryos at 40 hpf, PD184352 treated: n = 11 embryos at 40 hpf, DMSO treated: n = 13 embryos at 48 hpf, PD184352 treated: n = 8 embryos at 48 hpf). ANOVA followed by Tukey multiple comparison test. **d.** Quantification of 3D cavity shape/size descriptors (cavity profile length, cavity lateral aspect ratio, average cavity curvature) on AP orthogonal slices (2 independent experiments, DMSO treated: n = 30 embryos at 40 hpf, PD184352 treated: n = 33 embryos at 40 hpf, DMSO treated: n = 32 embryos at 48 hpf, PD184352 treated: n = 28 embryos at 48 hpf). ANOVA followed by Tukey multiple comparison test. **e.** Quantification of 3D cavity shape/size descriptors (cavity profile length, cavity lateral aspect ratio, average cavity curvature) on ML orthogonal slices (2 independent experiments, DMSO treated: n = 30 embryos at 40 hpf, PD184352 treated: n = 33 embryos at 40 hpf, DMSO treated: n = 32 embryos at 48 hpf, PD184352 treated: n = 28 embryos at 48 hpf). ANOVA followed by Kruskal-Wallis multicomparison test.

Supplementary Figure 7 Gordillo Pi et al.

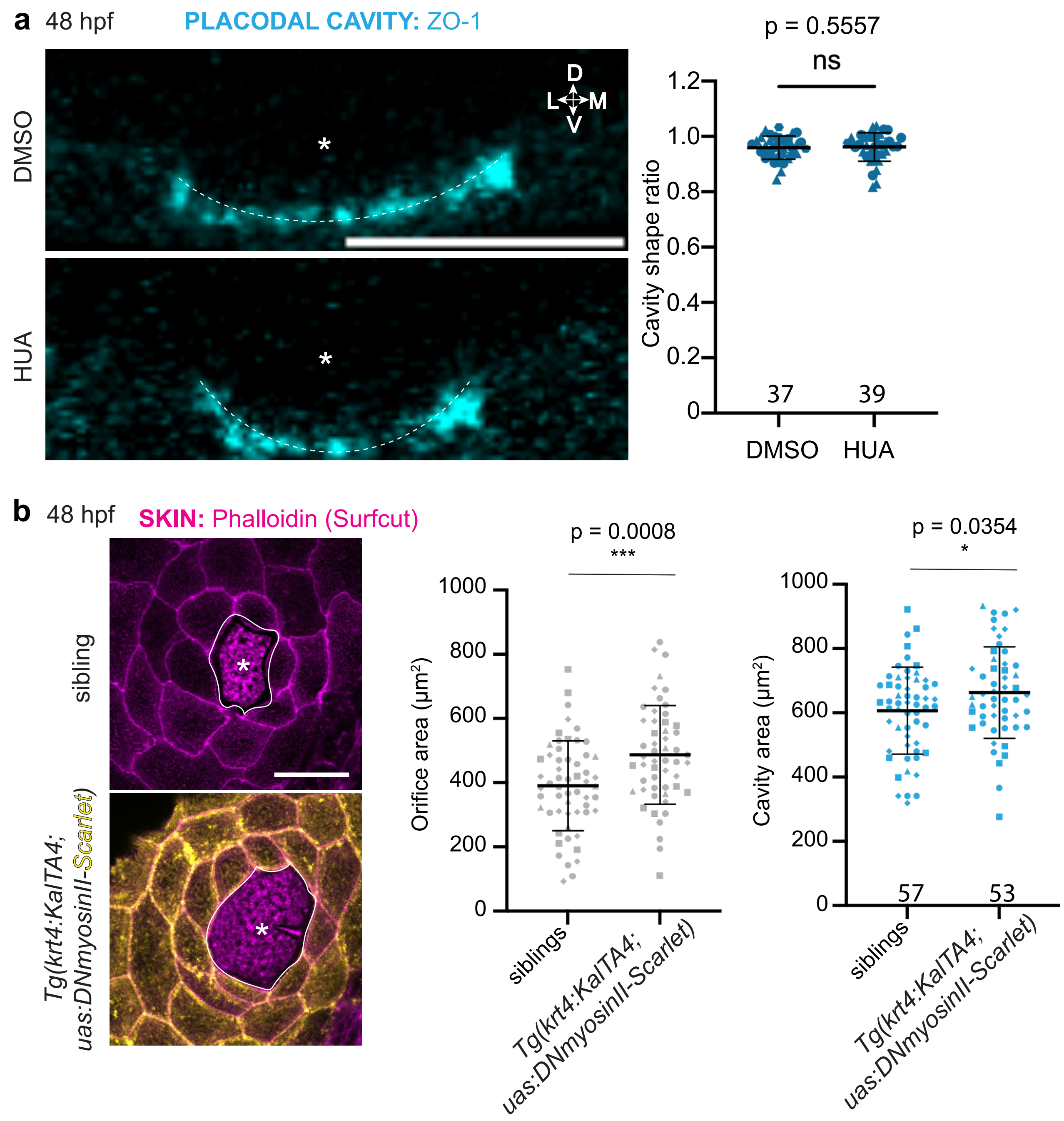

Supplementary Figure 7. Additional data on HUA treatment and inhibition of myosin II activity in the skin, related to Fig. 2 and Fig. 5**. a.** Left. Orthogonal ML sections of DMSO and HUA-treated embryos immunostained for ZO-1 (cyan) to reveal the cavity at 48 hpf. Dotted line = cavity profile ~~contour~~. Asterisk = orifice position. Scale bar = 20 µm. Right. Quantification of the cavity shape ratio (as defined in Fig. 5d') in HUA-treated embryos and DMSO-treated controls (3 independent experiments, n = 39 HUA-treated embryos, n = 37 DMSO controls). Mann-Whitney test. **b.** Images of 48 hpf *Tg(krt4:KalTA4; uas:DNmyosin-Scarlet*) double transgenic embryos and control siblings. Skin seen with phalloidin staining (magenta) extracted with the SurfCut plugin (see Methods). Scarlet visualised after DsRed immunostaining (yellow). White line = orifice. Asterisk = orifice. Scale bar = 20 µm. Quantification of orifice and cavity areas (4 independent experiments, n = 57 control siblings, n = 53 *Tg(krt4:KalTA4; uas:DNmyosin-Scarlet*) embryos). Unpaired, two-tailed t-test.
