# Supplementary material for: Dynamic interactions between epithelial skin cells and a sensory cavity sculpt the growing olfactory orifice
Source: Nat Commun. 2026 Jul 8;17:8452. doi: 10.1038/s41467-026-75307-y (PMC13478634; doi:10.1038/s41467-026-75307-y)
Supplement: Supplementary file 4 [file 41467_2026_75307_MOESM2_ESM.docx]

**Supplementary Movie legends**

**Supplementary Movie 1. 3D reconstruction of the orifice and cavity in a 48 hpf embryo**, related to Fig. 1**.** Peridermal skin cells labelled with the *Tg(krt5:Gal4-ERT; uas:RFP)* transgenic line (magenta) and cavity revealed by ZO-1 immunostaining (cyan). Arivis 3D reconstruction.

**Supplementary Movie 2. Live imaging of skin cell behaviors during the expansion of the olfactory orifice**, related to Fig. 2. Confocal live imaging (left) and segmentation (right) performed between 32 and 46 hpf on a *Tg(krt4:lynGFP)* embryo, in which the membranes of skin cells are labelled with GFP, showing the expansion of the olfactory orifice and concomitant remodeling (divisions, area and shape changes) of the skin cells located on the edge of the orifice (bordering cells). The arrowhead in magenta points to the orifice in the raw imaging data (left). See also Fig. 2a, a’. Maximum projection, dorsal view, anterior to the top and lateral to the left, delta t = 10 min. Scale bar: 20 µm.

**Supplementary Movie 3.** **Segmentation of the ZO-1-positive apical surfaces lining the cavity, and physical contact between the skin and the cavity**, related to Fig. 3. Arivis 3D reconstruction of a high resolution z-stack performed on a 48 hpf embryo immunostained for ZO1. The segmentation of the large, cuboidal apical surfaces lining the cavity is shown with colors in the 3D reconstruction, and the deep learning-based segmentation of the small, round apical tips is shown with yellow circles in the z-stack. For the z-stack, dorsal view, the first z-sections are the most dorsal and the z-stack progressively goes ventral, z-step of 0.36 µm. In the z-stack, cyan arrowheads indicate points of physical contact between skin/skin cell junctions and the upper part of the cavity.

**Supplementary Movie 4. Intercalation of olfactory placode neurons into the rosette,** related to Fig. 3. Confocal live imaging performed between 32 and 48 hpf on a *Tg(Xla.Tubb:DsRed)* embryo in which olfactory placode neurons are labelled (green). Arrows point to DsRed-positive neurons that intercalate in the rosette during the movie and send their dendrites towards the rosette center (i.e. towards the cavity). Maximum projection, dorsal view, anterior to the top and lateral to the left, delta t = 10 min. Scale bar: 20 µm.

**Supplementary Movie 5. Biphoton laser ablation of rosette placodal cells,** related to Fig. 3**.** 3D z-stacks performed on a *Tg(omp:meYFP); Tg(krt5:Gal4-ERT; uas:RFP)* embryo before and just after the ablation, at 32 hpf. The ablation targets a subset of meYFP*-*positive neurons forming the rosette. The green dot indicates the ablated region. The arrow points to the meYFP*-*positive axon bundle growing in the brain (reference[^77^](https://www.zotero.org/google-docs/?gut5MP)). Note that the skin is not affected by the ablation. Dorsal view, the first z-sections are the most dorsal and the z-stacks progressively go ventral, z-step of 1 µm. Scale bar: 20 µm.

**Supplementary Movie 6**. **3D z-stacks of F-actin and myosin II distribution,** related to Fig. 4. High resolution confocal z-stack performed on embryos carrying the *Tg(actb2:myl12.1-eGFP)* transgene to visualize non muscle myosin II (green) and labelled with phalloidin-rhodamine to reveal F-actin (magenta) at 32 and 48 hpf. White arrowheads indicate the actomyosin ring located on the uppermost region of the cavity (and between the MCCs and the neurons in the lateral part of the cavity at 48 hpf). The staining also allows to visualize cortical actomyosin and microridges in peridermal skin cells. Dorsal view, the first z-sections are the most dorsal and the z-stacks progressively go ventral, z-step of 0.31 µm.

**Supplementary Movie 7. Laser ablation of the myosin II ring located in the upper cavity,** related to Fig. 4. Example of a laser cut performed on the actomyosin ring of a 32 hpf *Tg(actb2:myl12.1-eGFP)* embryo, showing the immediate recoil after ablation. The laser cut is performed on the right part of the ring, at t5 (see the region of ablation depicted in Fig. 4b). Dorsal view, 1 z-section. Scale bar: 10 µm.

**Supplementary Movie 8**. **Live imaging of skin cell behaviors upon ERK inhibition,** related to Fig. 5**.** Confocal live imaging of a *Tg(krt4:lynGFP)* embryo (in which the membranes of skin cells are labelled with GFP) treated with the MEK inhibitor PD184352. Only the first 6 hours of the initial expansion phase are imaged, due to a drift of the embryo in the z direction. Maximum projection, dorsal view, anterior to the top and lateral to the left, delta t = 10 min. Scale bar: 20 µm.
